# Serum PINP, PIIINP, galectin-3, and ST2 as surrogates of myocardial fibrosis and echocardiographic left venticular diastolic filling properties

**DOI:** 10.3389/fphys.2015.00200

**Published:** 2015-07-13

**Authors:** E. Samuli Lepojärvi, Olli-Pekka Piira, Eija Pääkkö, Eveliina Lammentausta, Juha Risteli, Johanna A. Miettinen, Juha S. Perkiömäki, Heikki V. Huikuri, M. Juhani Junttila

**Affiliations:** ^1^Department of Internal Medicine, Medical Research Center Oulu, University of OuluOulu, Finland; ^2^Department of Radiology, Oulu University HospitalOulu, Finland; ^3^Department of Clinical Chemistry, Institute of Diagnostics, University of OuluOulu, Finland

**Keywords:** diastolic heart failure, fibrosis, biomarkers, magnetic resonance imaging, doppler echocardiography

## Abstract

**Objectives and Background:** Serum biomarkers have been proposed to reflect fibrosis of several human tissues, but their specific role in the detection of myocardial fibrosis has not been well-established. We studied the association between N-terminal propeptide of type I and III procollagen (PINP, PIIINP, respectively), galectin-3 (gal-3), soluble ST2 (ST2), and myocardial fibrosis measured by late gadolinium enhanced cardiac magnetic resonance imaging (LGE CMR) and their relation to left ventricular diastolic filling properties measured by tissue Doppler echocardiography (E/e') in patients with stable coronary artery disease (CAD).

**Methods and Results:** We determined the PINP, PIIINP, gal-3, and ST2 serum levels and performed LGE CMR and echocardiography on 63 patients with stable CAD without a history of prior myocardial infarction. Myocardial late gadolinium enhancement T1 relaxation time was defined as a specific marker of myocardial fibrosis. ST2, PINP, and PIIINP did not have a significant correlation with the post-LGE T1 relaxation time tertiles (NS for all), but the lowest post-LGE T1 relaxation time tertile had significantly higher gal-3 values than the other two tertiles (*p* = 0.002 and 0.002) and higher E/é-values (*p* = 0.009) compared to the highest T1 relaxation time tertile. ST2 (*p* = 0.025 and 0.029), gal-3 (*p* = 0.003 and < 0.001) and PIIINP (*p* = 0.001 and 0.007) levels were also significantly higher in the highest E/é tertile, compared to the other two tertiles.

**Conclusions:** Elevated serum levels of gal-3 reflect the degree of myocardial fibrosis assessed by LGE CMR. Gal-3, ST2, and PIIINP are also elevated in patients with impaired LV diastolic function, suggesting that these biomarkers are useful surrogates of structural and functional abnormality of the myocardium.

## Introduction

Impaired left ventricular (LV) diastolic filling in subjects with preserved LV function has been associated with worse outcome among coronary artery disease (CAD) patients and patients with heart failure with preserved LV function (Rusinaru et al., 2014). The etiological background for impaired LV diastolic filling properties is not well-known, but one possible underlying mechanism could be the accumulation of diffuse fibrosis in the myocardium. Diffuse interstitial fibrosis can be assessed with cardiovascular magnetic resonance imaging (CMR) by using late gadolinium enhancement (LGE). Relaxation time T1 mapping has been shown to correlate with interstitial fibrosis measured from endomyocardial biopsies or with invasively measured left ventricular stiffness (Iles et al., 2008; Miller et al., 2013; Ellims et al., 2014). Many biomarkers of fibrosis have also been proposed to reflect myocardial fibrosis. Biomarkers related to fibrosis, such as N-terminal propeptide of type I and III procollagens (PINP and PIIINP, respectively), galectin-3 (gal-3), and soluble ST2 protein (ST2) have been associated with poor outcome among heart failure patients (Cicoira et al., 2004; Pascual-Figal et al., 2009; Velagaleti et al., 2010; De Boer et al., 2011; Bayes-Genis et al., 2012; Lok et al., 2013). Although these biomarkers may have predictive value, their ability to detect myocardial interstitial fibrosis is not well-established.

The aim of this study was to determine the association of fibrosis biomarkers and myocardial interstitial fibrosis measured by LGE CMR and their relation to left ventricular diastolic filling measured by tissue Doppler echocardiography in patients with stable coronary artery disease (CAD).

## Materials and methods

### Patient population

Sixty-three consecutive patients with angiographically documented stable CAD were prospectively recruited from the ARTEMIS-Oulu database (Cardiovascular Complications in Type II Diabetes Study; registered at ClinicalTrials.gov, Record 1539/31/06, Identifier NCT01426685). The exclusion criteria included rhythm other than sinus rhythm, reduced left ventricular ejection fraction, greater than mild valvular disease or previous valve surgery, clinical history of myocardial infarction (Q-waves in ECG, myocardial scar, or segmental wall motion abnormalities seen in echocardiography), permanent pacemaker, significant renal disease, and claustrophobia. The study was approved by the local institutional ethics committee. Written informed consent was obtained from all the patients.

### Biomarkers

The concentrations of gal-3 and ST2 were determined from serum samples. Serum was prepared by allowing the blood to clot for 30 min followed by centrifugation at 2000 ×g for 10 min. The serum was stored at -20°C until analyzed. ST2 levels were analyzed using a sandwich, enzyme-linked immunosorbent assay (ELISA) (Human ST2/IL-1 R4 Quantikine ELISA, R&D Systems Inc., Minneapolis, MN) with a sensitivity of 5.1 pg/mL. Gal-3 levels in serum were determined by an enzyme-linked immunosorbent assay (ELISA) from BG Medicine (Waltham, MA, USA). The limit of detection (LoD) for the assay was 1.13 ng/mL (Christenson et al., 2010).

Serum Intact PINP concentrations (Koivula et al., 2010) were analyzed with the automated iSYS instrument (IDS, Newcastle, U.K) using 20 μL samples and serum PIIINP (Risteli et al., 1988) concentrations with specific radioimmunoassays (Orion Diagnostica, Espoo, Finland) using 200 μL or 100 μL samples, respectively.

### Echocardiography

A thorough transthoracic echocardiographic evaluation was made utilizing the same General Electric Vivid seven for all patients. Parasternal long axis view and M-mode were used to obtain left ventricular (LV) diameters and wall thickness. LV mass was derived from the ASE equation. LV ejection fraction was measured from the apical view by the biplane method from the 2- and 4-chamber views. Left ventricular diastolic filling was assessed by end-expiration ratio of peak early diastolic mitral velocity to tissue Doppler-derived peak early diastolic mitral annular velocity measured in the septal mitral annulus (E/é).

### Late gadolinium enhancement CMR

CMR with the same 1.5T scanner (GE Optima MR450w, GE Healthcare, Milwaukee, WI) and 32-channel cardiac coil was performed on all the patients. Mid-ventricular short axis images were obtained. T1 was measured i.e., T1 mapping was performed with a ECG-triggered Look-Locker sequence (TR 4440 s, TE 2.016 ms, 30 TI's between 95 and 1279 ms, in-plane resolution 1.48 mm, slice thickness 8 mm). The images were acquired during breath holds. MRI images were analyzed off-line using an in-house MATLAB application (Matlab, MathWorks Inc, Natick, MA). Regions of interest were segmented manually. The segments with visible LGE i.e., replacement fibrosis were excluded from the analysis. The possible position differences between images with different TI's were taken into account by segmenting each image separately and calculating ROI-wise T1 using the mean intensities of each image. T1 relaxation time was measured 10–15 min after contrast agent injection (0.4 mL/kg but not more than 30 mL of Dotarem (500 mM, Guerbet AG, Zürich, Switzerland).

### Statistical analysis

All continuous data are presented as mean ± standard deviation. IBM® SPSS® Statistics v. 21 (IBM Corp., Armonk, NY) was used to analyze all statistics. Because of the skewed distribution of the variables, no correlation co-efficient were analyzed. For the same reason and due to the small study population we tested differences between tertiles divided according to LGE T1 relaxation time and E/é. Oneway analysis of variance was used followed by post hoc Bonferroni test in comparisons between the tertiles. All results with *p* < 0.05 were considered statistically significant.

## Results

The mean T1 relaxation time was 365 (±43.9) ms, the mean value of E/é 9.77 (±4.92) and the concentration of ST2 was 20.37 (±6.62) ng/mL, of gal-3 12.13 (±4.93) ng/mL, of PINP 36.27 (±16.29) ng/mL, and that of PIIINP 3.81 μg/L (±1.13).

Age, gender, BMI, diabetes, history of smoking, medication, blood pressure, left ventricular ejection fraction, LV mass, and LV mass index, Syntax score, renal function, HbA1c levels, serum cholesterol levels, high sensitive CRP, and BNP levels did not differ significantly between the T1 relaxation time tertiles (Table 1).

**Table 1:** **Characteristics of patients divided according to the amount of fibrosis measured by magnetic resonance imaging**.

	**1st**	**2nd**	**3rd**	***p*-value**
Age (years)	68.7±8	67.8±8.8	64.6±7.0	0.27
BMI	28.1±4.2	28.0±4.9	27.8±3.2	0.97
SBP/DBP (mmHg)	155∕82±21∕13	145∕80±27∕10	147∕82±21∕9	0.29/0.75
EF (%)	66%±7	63%±5	65%±6	0.16
LV mass	236±58	252±77	211±30	0.13
LV mass index	123±27	131±35	112±15	0.12
S-creatinine (μmol/L)	87±26	77±18	73±16	0.47
S-Cholesterol	4.1±1.1	3.8±0.8	4.0±0.9	0.73
S-LDL-Cholesterol	2.4±0.9	2.3±0.7	2.2±0.9	0.83
B-HbA1c (%)	6.6±1.0	6.4±1.0	6.1±0.7	0.29
BNP (ng/L)	91±76	73±71	59±65	0.77
hs-CRP (mg/L)	3.7±9.1	2.9±5.4	1.7±2.1	0.55
Syntax score	2.9±4.4	6.9±11.2	3.7±5.7	0.29
Smoking (ongoing/ex)	10%∕52%	14%∕55%	5%∕50%	0.86
Type II diabetes	76%	77%	75%	0.99
Sex (male)	57%	41%	50%	0.57
Medication				
B-blocker	86%	86%	80%	0.83
ACEi/ARB	71%	68%	50%	0.31
CCB	24%	14%	5%	0.23
Diuretics	43%	18%	40%	0.17
Statins	91%	91%	90%	0.99

*BMI, body mass index; BP, blood pressure; EF, ejection fraction; LV, left ventricle; LDL, low-density lipoprotein; HbA1c, Glycosylated Hemoglobin, Type A1C; BNP, brain natriuretic peptide; hs-CRP, high sensitive C-reactive protein; ACEi, angiotensin converting enzyme inhibitor; ARB, angiotensin II receptor blocker; CCB, calcium channel blocker*.

**Figure 1:**
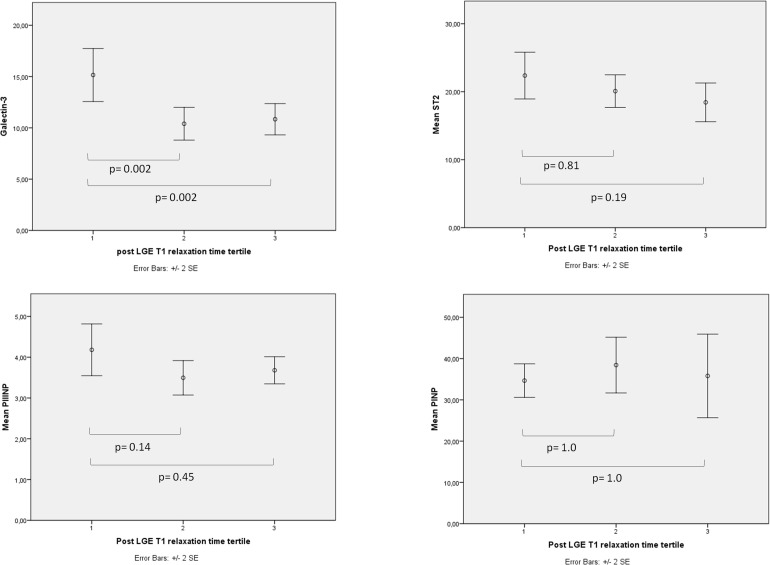
**The correlations between LGE T1 relaxation time and biomarkers**. When subjects were divided into tertiles according to LGE T1 relaxation time (1st tertile had the shortest LGE T1 relaxation time and the highest amount of interstitial fibrosis) Galectin-3 was the only biomarker of fibrosis that was significantly higher in the 1st tertile compared to the other two tertiles.

Subjects in the shortest post-LGE T1 relaxation time tertile had significantly higher gal-3 levels compared to the other two tertiles (15.2 ± 5.9 ng/mL vs. 10.4 ± 3.7 ng/mL, *p* = 0.002 between tertiles 1 vs. 2 and 15.2 ± 5.9 vs. 10.4 ± 2.8, *p* = 0.002 between 1 vs. 3). However, there was no significant difference in ST2 (22.4 ± 7.7 ng/mL vs. 20.1 ± 5.5 ng/mL, *p* = 0.81 and 22.4 ± 7.7 vs. 18.4 ± 6.4, *p* = 0.19), PIIINP (4.2 ± 1.5 μg/L vs. 3.5 ±1.0 μg/L, *p* = 0.14 and 4.2 ± 1.5 μg/L vs. 3.7 ± 0.7 μg/L, *p* = 0.45), or PINP (34.7 ± 9.3 ng/mL vs. 38.4 ± 15.5 ng/mL, *p* = 1.0 and 34.7 ± 9.3 ng/mL vs. 35.8 ± 22.6 ng/mL, *p* = 1.0) between the LGE T1 tertiles (Figure 1).

**Figure 2:**
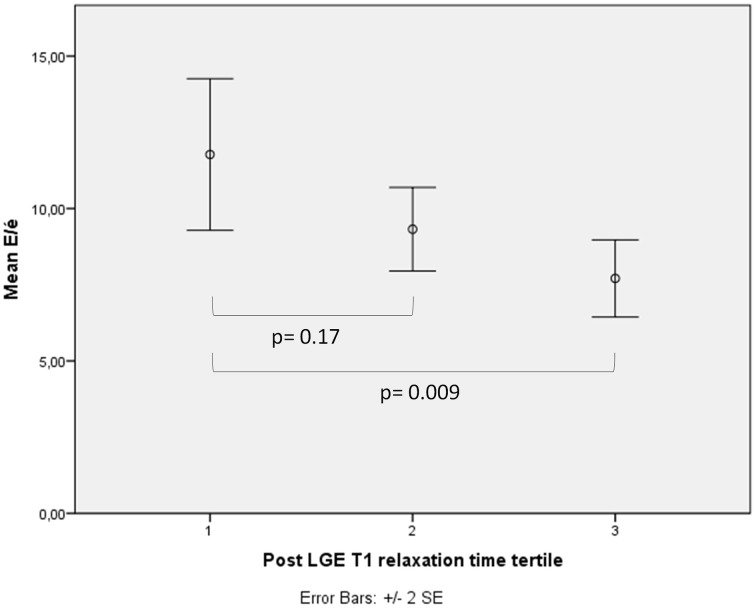
**The correlation between LGE T1 relaxation time and diastolic function**. When subjects were divided into tertiles according to LGE T1 relaxation time (1st tertile had the shortest LGE T1 relaxation time and the highest amount of interstitial fibrosis) the 1st tertile had significantly more impaired LV diastolic function as measured by E/é-values compared to the 3rd tertile.

**Figure 3:**
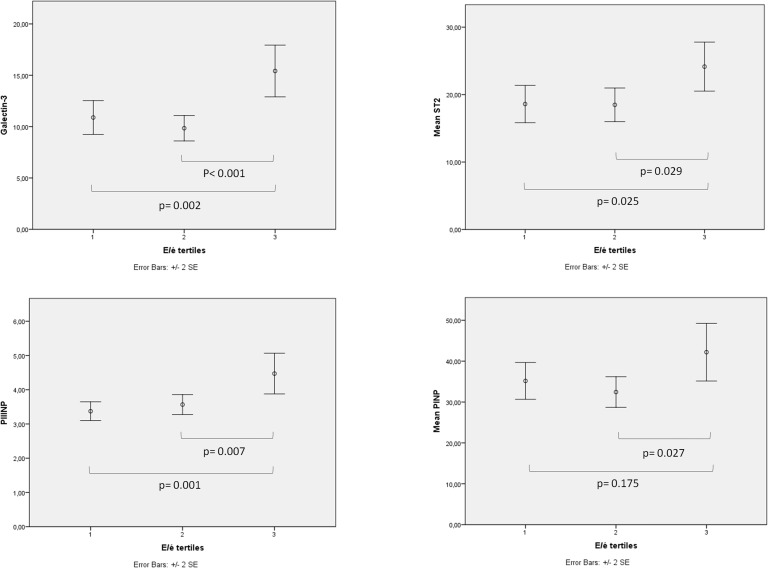
**The correlation between diastolic function and biomarkers**. When subjects were divided into tertiles according to LV diastolic function (E/é-values) the tertile with the most impaired LV diastolic function had significantly higher serum levels of GAL-3, ST2, and PIIINP compared to the other two tertiles.

Higher E/é-values in the shortest post-LGE relaxation time tertile were also observed compared to the longest relaxation time tertile (11.8 ± 5.7 vs. 7.7 ± 2.8, *p* = 0.009) (Figure 2). The subjects in the highest E/é tertile had significantly higher serum levels of gal-3 (10.9 ± 4.1 ng/mL vs. 15.4 ± 5.2 ng/mL, *p* = 0.002 between tertiles 1 vs. 3 and 9.8 ± 2.8 ng/mL vs. 15.4 ± 5.2 ng/mL, *p*<0.001 between tertiles 2 vs. 3), ST2 (18.6 ± 6.6 ng/mL vs. 24.2 ± 7.3 ng/mL, *p* = 0.025 and 18.5 ± 5.4 ng/mL vs. 24.2 ± 7.3 ng/mL, *p* = 0.029), and PIIINP (3.4 ± 0.8 μg/L vs. 4.5 ± 1.8 μg/L, *p* = 0.001 and 3.6 ± 0.8 μg/L vs. 4.5 ± 1.8 μg/L, *p* = 0.007) (Figure 3).

## Discussion

### Main findings

Gal-3 was the only serum biomarker that had a significant correlation to diffuse myocardial fibrosis estimated by LGE CMR T1 mapping in patients with stable CAD. Elevated levels of other biomarkers, such as ST2 and PIIINP, had no significant association to fibrosis, but these biomarkers were associated with impaired left ventricular filling assessed by tissue Doppler echocardiography. Furthermore, LGE T1 relaxation time was closely associated with E/é showing that diffuse myocardial fibrosis is an important determinant of cardiac diastolic function in patients with uncomplicated stable CAD. Myocardial fibrosis had no significant relationship with any demographic variable, left ventricular systolic function, severity of CAD (Syntax score), or any metabolic risk variable.

### Cardiac MRI

CMR has emerged as a non-invasive imaging method for focal fibrosis but it also allows the assessment of diffuse interstitial fibrosis. Isolated post-contrast T1 relaxation time has been shown to have a strong correlation with histologically confirmed myocardial fibrosis in small studies involving patients with heart failure (Iles et al., 2008; Miller et al., 2013). T1 relaxation time has also some correlation with echocardiographic markers of impaired diastolic function such as septal E' and E/é in patients with diabetes but no underlying CAD as a marker of so-called diabetic cardiomyopathy (Jellis et al., 2011; Ng et al., 2012). One study including patients with ischemic cardiomyopathy also showed a correlation between E/é and visually estimated LGE (Raman et al., 2009). Isolated post-contrast T1 relaxation time was also the only variable that correlated with invasively measured LV stiffness after multivariate analysis in cardiac transplant recipients (Ellims et al., 2014).

Previous studies using T1 relaxation time analysis to estimate the amount of fibrosis have excluded patients with stable uncomplicated CAD. In our study we were able to show that also in patients with CAD, isolated post-contrast T1 relaxation time correlates with diastolic filling properties, even when areas of visible LGE where excluded from analysis. In post hoc analysis we could show that the patients in the tertile with the highest amount of interstitial fibrosis in CMR had significantly higher E/é-values than the tertile with least fibrosis.

### Biomarkers

Myocardial collagen tissue consists mostly of collagen type I and III. Procollagen N-terminal peptides, which can be measured from blood samples, have been used as surrogates of myocardial fibrosis. Nevertheless there has been some controversial evidence of PINP and PIIINP as biomarkers of collagen biosynthesis and as predictors of outcome of heart failure patients. Gal-3 and ST2 are more novel biomarkers of fibrosis. Gal-3 is an important mediator that induces fibroblasts to proliferate and deposit collagen, which contributes to myocardial fibrosis and remodeling. ST2 is a member of the interleukin receptor family and the gene expression of ST2 is upregulated in fibroblasts and cardiomyocytes subjected to mechanical stress. ST2 also prevents the IL-33 effects in reducing fibrosis and hypertrophy. GAL-3 (Lok et al., 2010, 2013; De Boer et al., 2011; Ho et al., 2012; Lopez-Andrés et al., 2012) and ST2 (Pascual-Figal et al., 2009; Manzano-Fernandez et al., 2011) have been shown to have predictive value for adverse cardiac events and mortality especially in patients with heart failure and ST2 also in patients after acute coronary syndrome (Eggers et al., 2010).

In this study we found a correlation between the LGE T1 relaxation time and GAL-3, but the other biomarkers of fibrosis did not have a significant correlation to myocardial fibrosis measured by LGE CMR. One of the reasons for these observations might be that ST2 concentrations are thought to raise as a result of increased myocardial strain whereas gal-3 can be seen as an initiator of collagen deposition and therefore as a better surrogate of incipient or existing interstitial fibrosis even without systolic or diastolic impairment.

In the tertile analysis divided according to diastolic function i.e., E/é-values, we were able to show significant differences in the biomarker levels of Gal-3, ST2, and PIIINP between the most marked diastolic impairment tertile compared to the other two tertiles. These data provide evidence of the utility of these serum biomarkers in the rapid diagnosis of cardiac diastolic dysfunction, but these findings need confirmation in larger patient samples.

### Limitations

The relatively small sample size of the study prevents definite conclusions regarding the lack of correlation between some of the biomarkers, such ST2 and PIIINP, and myocardial fibrosis measured by CMR. Measurement of relaxation time T1 by the LGE method may also have some limitations in terms of reliable quantification of diffuse myocardial fibrosis. Despite these limitations, we feel that the present findings provide some useful information about the value of serum biomarkers as predictors of both myocardial fibrosis and cardiac diastolic properties.

## Conclusion

In patients with uncomplicated CAD, serum biomarkers, especially gal-3, are associated with diffuse interstitial fibrosis imaged with cardiac MRI. Additionally, these biomarkers are associated with echocardiographically measured impaired LV diastolic filling properties. These results suggest that LV interstitial fibrosis plays an important role in impaired diastolic function among CAD patients and the level of cardiac diastolic dysfunction can be assessed with serum biomarkers of fibrosis.

## Conflict of interest statement

The authors declare that the research was conducted in the absence of any commercial or financial relationships that could be construed as a potential conflict of interest.

## Abbreviations

LV: left ventricle
EF: ejection fraction
CAD: coronary artery disease
CMR: cardiovascular magnetic resonance
LGE: late gadolinium enhancement
PINP: N-terminal propeptide of type I procollagen
PIIINP: N-terminal propeptide of type III procollagen
Gal-3: galectin-3
ST2: soluble ST2 protein
ELISA: enzyme-linked immunosorbent assay
TR: time to repetition
TE: time to echo
TI: inversion time
BMI: body mass index
GHbA1c, Glycosylated Hemoglobin: Type A1C
BNP: brain natriuretic peptide
hs-CRP: high sensitive C-reactive protein.
